# Association between intracellular adenosine triphosphate content of CD4^+^ T lymphocytes and mortality in sepsis patients: A prospective observational study

**DOI:** 10.1002/iid3.1286

**Published:** 2024-06-11

**Authors:** Ying Xian, Dan Xie, Jian Zhu, Changlong Zheng, Min Fan, Kefeng Jiang, Kouxing Zhang

**Affiliations:** ^1^ Department of General Intensive Care Unit, Lingnan Hospital The Third Affiliated Hospital of Sun Yat‐Sen University Guangzhou People's Republic of China; ^2^ Department of Emergency Intensive Care Unit The Third Affiliated Hospital of Sun Yat‐Sen University Guangzhou People's Republic of China; ^3^ Department of Parasitology, Zhongshan School of Medicine Sun Yat‐Sen University Guangzhou People's Republic of China

**Keywords:** CD4^+^ T lymphocytes, immune function, intracellular adenosine triphosphate (iATP), mortality, prognosis, sepsis

## Abstract

**Objective:**

This study aimed to link intracellular adenosine triphosphate content in CD4^+^ T lymphocytes (CD4^+^ iATP) with sepsis patient mortality, seeking a new predictive biomarker for outcomes and enhanced management.

**Methods:**

61 sepsis patients admitted to the Intensive Care Unit between October 2021 and November 2022 were enrolled. iATP levels were gauged using whole blood CD4^+^ T cells stimulated with mitogen PHA‐L. Based on CD4^+^ iATP levels (<132.24 and ≥132.24 ng/mL), patients were categorized into two groups. The primary endpoint was all‐cause mortality. To identify factors associated with mortality, both univariate and multivariate Cox proportional hazard analyses were conducted.

**Results:**

Of the patients, 40 had high CD4^+^ iATP levels (≥132.24 ng/mL) and 21 had low levels (<132.24 ng/mL). In a 28‐day follow‐up, 21 (34.4%) patients perished. Adjusting for confounders like SOFA score, APACHE II score, lactic acid, and albumin, those with low CD4^+^ iATP had three‐ to fivefold higher mortality risk compared to high CD4^+^ iATP patients (61.9% vs. 20.0%; hazard ratio [95% confidence interval], Model 1: 4.515 [1.276–15.974], *p* = .019, Model 2: 3.512 [1.197–10.306], *p* = .022). CD4^+^ iATP correlated positively with white blood cell and neutrophil counts but not with lymphocytes, CD3, and CD4 counts.

**Conclusions:**

Low CD4^+^ iATP levels were associated with a higher risk of mortality in sepsis patients. Measurement of CD4^+^ iATP may serve as a useful tool for identifying patients at a higher risk of mortality and could potentially provide a basis for clinical treatment. Further research is warranted to fully elucidate the underlying mechanisms of this association.

## Graphical abstract

This study found that low CD4^+^ iATP levels were associated with a higher risk of mortality in sepsis patients. Measurement of CD4^+^ iATP may serve as a useful tool for identifying patients at a higher risk of mortality and could potentially provide a basis for clinical treatment. Further research is warranted to fully elucidate the underlying mechanisms of this association.

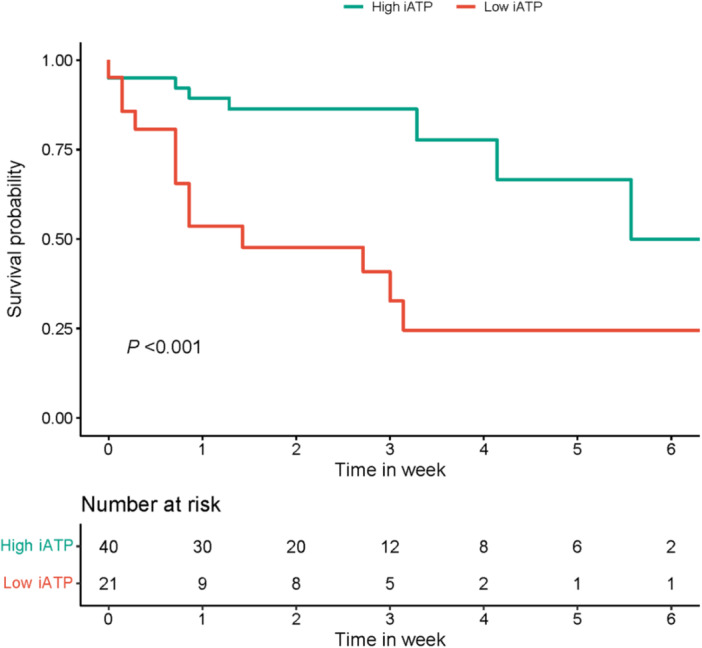

## 1 INTRODUCTION

Sepsis, characterized by a systemic inflammatory response syndrome resulting from infection, remains a significant health concern with ICU mortality rates ranging from 6% to 30%, escalating to over 40% in patients with septic shock.^1^, ^2^ Currently, there is a lack of reliable prognostic indicators for critically ill patients with infections, leading to a dearth of targeted interventions and treatment strategies. Commonly employed prognostic assessment methods, such as the Sequential Organ Failure Assessment (SOFA) and Acute Physiology and Chronic Health Evaluation Score II (APACHE II), suffer from subjectivity and fail to provide objective and intuitive data.^3^, ^4^ Therefore, establishing an improved prognostic warning mechanism for critically ill patients with infections has emerged as a significant area of research.

The immune system plays a crucial role in sepsis, and mounting evidence suggests the occurrence of both hyperinflammatory and immunosuppressive responses in septic patients.^5^, ^6^, ^7^ Apart from the initial hyperinflammatory response, a considerable number of sepsis patients develop sepsis‐associated immunosuppression, which has been implicated in disease progression and adverse outcomes,^6^, ^8^, ^9^ this immunosuppression is characterized by dysfunctional T cell responses, including apoptosis,^10^ exhaustion,^11^ and impaired effector function,^12^ further exacerbating the disease's severity. Several biomarkers, including lymphocyte counts, CD4^+^ cell counts, monocyte human leukocyte antigen DR (mHLA‐DR), programmed death receptor‐1 (PD‐1), B and T lymphocyte attenuator (BTLA), neutrophil chemotaxis activity, and endogenous cytokines, have been proposed to monitor immune status and predict outcomes in sepsis.^13^, ^14^, ^15^, ^16^, ^17^ Mitochondrial dysfunction is a characteristic feature of immune suppression in sepsis,^18^ and it is linked to organ failure in sepsis.^19^, ^20^, ^21^ However, these biomarkers have certain limitations. Lymphocyte counts, while readily accessible, lack specificity due to various confounding factors. The detection of mHLA‐DR is intricate, and the lack of standardized protocols and thresholds across studies hinders result comparability and clinical applicability.^22^, ^23^, ^24^ Immunomodulators may improve immune function and improve prognosis, but the curative effect is not clear due to lack of good monitoring indicators. Thus, further research is necessary to identify and validate biomarkers capable of accurately monitoring immune status, predicting sepsis outcomes, and feasible for clinical implementation.

The CD4+ cell ATP release assay evaluates the level of intracellular adenosine triphosphate (iATP) in purified CD4^+^ T lymphocytes after being stimulated with phytohemagglutinin‐L (PHA‐L). This technique has been developed to evaluate cell‐mediated immune function, forecast the risk of infection, and monitor the rejection of solid organ transplants.^25^, ^26^ Regarding severe infections, certain studies have indicated that within the initial 24 h of ICU admission, there is a notable difference in iATP levels within CD4^+^ T lymphocytes between survivors and non‐survivors. The former group displays higher levels according to those studies.^26^, ^27^ However, no further analysis was carried out on its correlation with the absolute counts of CD4^+^ T lymphocytes and its predictive value on the risk of all‐cause mortality. The objective of this study is to investigate the prognostic significance of CD4^+^ T lymphocyte iATP levels in critically ill patients with infections. Additionally, it aims to examine the relationship between elevated ATP levels and the outcomes of infection. The findings of this research hold promise in contributing new insights toward establishing a prognostic warning mechanism for critically ill patients with infections in clinical practice.

## 2 METHODS

### 2.1 Study population

**Figure 1:**
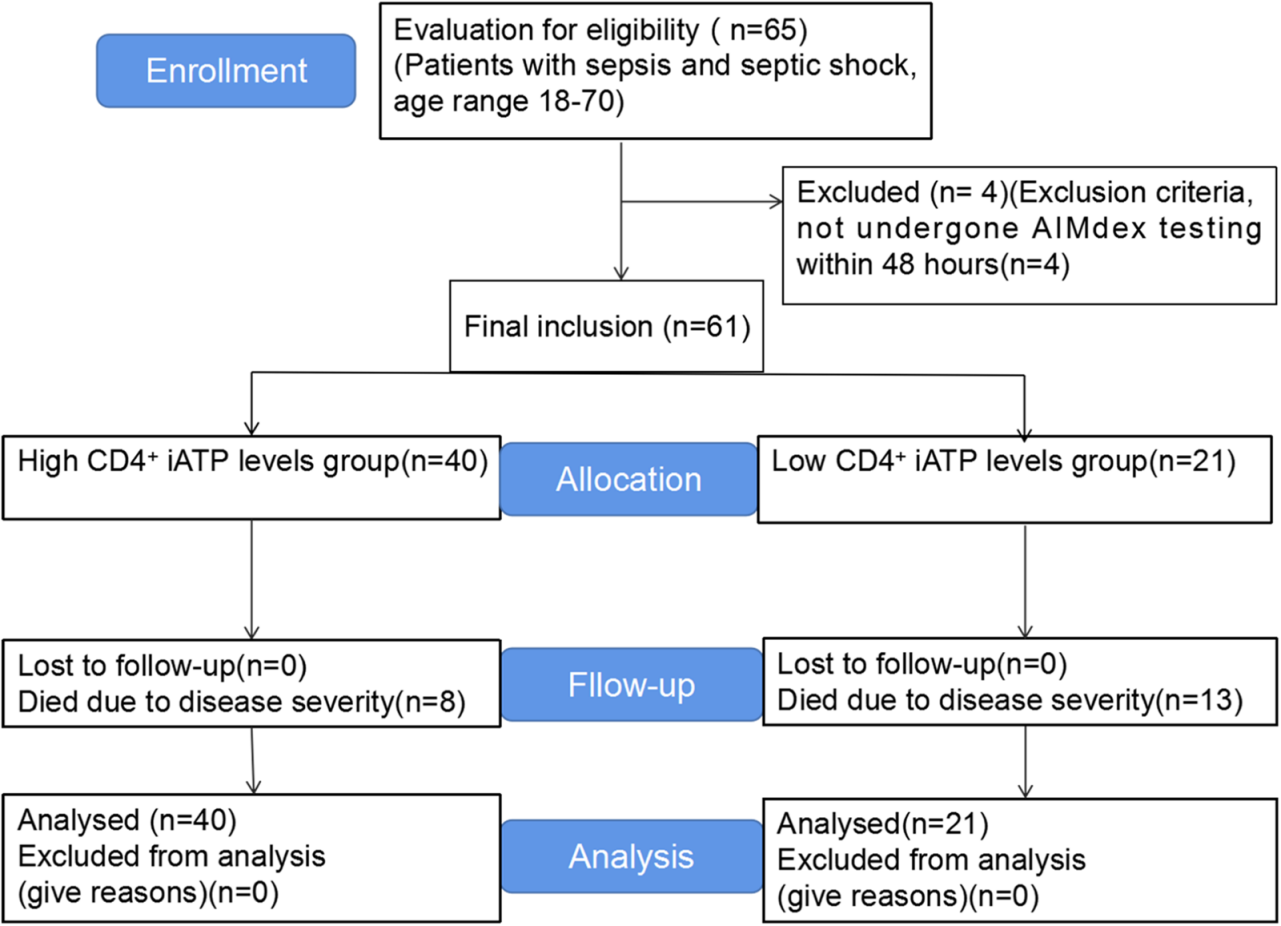
The study flow diagram. iATP, intracellular adenosine triphosphate.

A total of 65 consecutive sepsis patients who met the criteria were admitted to the intensive care unit (ICU) of The Third Affiliated Hospital of Sun Yat‐sen University between October 2021 and November 2022. Among them, four patients were excluded due to the lack of CD4^+^ iATP results. Finally, a total of 61 sepsis patients were included in this study. Based on their CD4^+^ iATP levels, they were divided into a high CD4^+^ iATP group and a low CD4^+^ iATP group (CD4^+^ iATP <132.24 ng/mL and ≥132.24 ng/mL). Throughout the follow‐up period, there were no dropouts among the patients (Figure 1). Sepsis was defined according to the consensus established by the Third International Task Force for Sepsis and Septic Shock (Sepsis‐3 definition).^28^, ^29^ The diagnosis of sepsis was established through a comprehensive evaluation that considered all accessible information, such as imaging results, response to antibiotics, and surgical findings. Inclusion criteria:patient age range 18‐70 years with sepsis at the time of admission; SOFA ≥ 2; patients with measurements of CD4^+^ iATP within 48 h of admission. Patients younger than 18 years; pregnant and lactating patients; patients with autoimmune diseases or malignant tumors under immunosuppressive therapy; patients without measurements of CD4^+^ iATP; patients having uncontrolled hemorrhage or other progressive diseases who were not expected to survive 24 h and patients who died within 24 h were excluded. This study followed the principles established in the Declaration of Helsinki and received approval from the Ethics Committee of The Third Affiliated Hospital of Sun Yat‐sen University (No. [2022]02‐216‐01). Written informed consent was obtained from all participants, allowing the utilization of their data for research purposes.

### 2.2 Data collection

Baseline data, including demographic characteristics, comorbidities, results of laboratory tests, treatment methods, and medications prescribed at discharge, were obtained from the electronic clinical management system. Comorbidities were ascertained based on preadmission diagnoses or diagnoses made during hospitalization. Laboratory examination records, including CD4^+^ iATP levels, were derived from the initial collection of whole blood samples obtained upon admission to the ICU.

### 2.3 Measurement of CD4^+^ iATP levels

CD4^+^ T cell iATP levels were measured using the luciferin‐luciferase reaction, in accordance with the manufacturer's instructions (Immune cell function assay kit, AIMdex^R^, Guangzhou Leide Biosciences Co., Ltd.). To elaborate, 100 μL of whole blood was diluted fourfold and then incubated with 25 μL of PHA‐L (8.75 ng/mL) for a time period of 15–18 h inside a 5% carbon dioxide incubator set at 37 ± 0.5°C. Following this, CD4^+^ T cells were isolated and purified from the aforementioned solution utilizing magnetic beads that had been conjugated with monoclonal antibodies specific to CD4^+^ T cells. To extract the iATP, a lysis buffer was added to the CD4^+^ T cell sample. The resulting cell lysate was thoroughly mixed with a luciferin/luciferase mixture, and the bioluminescent product was measured after a 5‐min reaction using a luminometer (JR‐I, Weihai Wego Biotech Co., Ltd.). To establish an iATP standard curve, various concentrations (0, 50, 100, 200, 400, and 800 ng/mL) of iATP calibrators were employed. A comparison was made between the iATP levels of CD4^+^ T cells‐depleted and non‐depleted samples to ensure that the readings were exclusively attributable to iATP produced by the isolated cells.

### 2.4 Endpoint and clinical definition

The primary endpoint was 28 days of all‐cause mortality. SOFA scores were employed to evaluate organ dysfunction in ICU patients.^30^ The SOFA scores were designed specifically for this purpose. Additionally, the APACHE II score, comprising age, the Chronic Health Index, and the Acute Physiology Score (APS), was used. The APS was derived from 12 physiological parameters, including vital signs, arterial blood gas measurements, laboratory results, the Glasgow Coma Scale, among others. The calculation of the APACHE II score followed the methodology described by Knaus et al.^31^

### 2.5 Statistical analysis

Patients were categorized into two groups based on their CD4^+^ iATP levels: iATP <132.24 ng/mL and iATP ≥132.24 ng/mL, determined as the optimal cut‐off value (Supporting Information S1: Figure 1). The optimal cutoff value was determined by assessing the ROC curve to achieve the best balance between sensitivity and specificity. Normally distributed continuous variables were described as means ± standard deviation (SD) and compared using Student's *t* test. Non‐normally distributed continuous variables were reported as medians (interquartile ranges [IQRs]) and were compared using Mann–Whitney *U* test. Categorical variables were expressed as numbers (percentages) and the chi‐squared test was used for categorical variables. Correlations between CD4^+^ iATP and other immune‐related parameters were assessed using the Spearman correlation test. The proportional hazards assumption was examined using Schoenfeld residuals. Time‐to‐event data were visualized using Kaplan–Meier curves, and survival between groups was compared using log‐rank tests. Univariate and multivariate analyses were conducted using Cox proportional hazard regression to identify significant predictors of mortality. Model 1 included CD4^+^ iATP, APACHE II score, SOFA score, lactic acid, albumin level, and presence of septic shock, while Model 2 included CD4^+^ iATP, age, gender, and SOFA score and the application of CRRT. A significance level of two‐sided *p* ≤ .05 was considered statistically significant for all analyses. SPSS 20.0 software and R software (version 4.2.2) were used for statistical analysis.

## 3 RESULTS

### 3.1 Baseline characteristics

The baseline characteristics stratified by CD4^+^ iATP categories (CD4^+^ iATP <132.24 ng/mL and ≥132.24 ng/mL) are presented in Table 1. Among the patients, 40 had high levels of CD4^+^ iATP (mean age 61.95 ± 15.69 years, 22.50% female), while 21 had low levels of CD4^+^ iATP (mean age 51.62 ± 16.68 years, 4.76% female). However, Compared to patients with high CD4^+^ iATP levels, those with low CD4^+^ iATP levels had a lower prevalence of hypertension (45.00% vs. 14.29%, *p* = .016), more CRRT treatment (27.50% vs. 57.15%, *p* = .023), higher SOFA scores (11.00 [8.00–16.00] vs. 7.00 [4.75–10.25], *p* = .003), lower CD8 counts (0.07 [0.02–0.20] vs. 0.20 [0.11–0.39], *p* = .014), lower platelets level (62.00 [30.00–103.00] vs. 182.00 [113.50–238.50], *p* < .001), lower CD4^+^ iATP levels (61.58 [42.35–97.69] vs. 325.54 [219.84–498.80], *p* < .001), lower CD4^+^iATP/CD4^+^ T cell count (889.53 ± 886.91 vs. 1666.27 ± 1217.75, *p* = .023) and higher shock rate (66.67% vs. 30.00%, *p* = .006). There were no significant differences observed between the two groups in terms of gender, APACHE II scores, diabetes mellitus, lactate level (Lac), albumin levels, lymphocyte counts, CD4^+^ T cell count, IL‐6, and other laboratory examinations. Supporting Information S1: Table 1 provides baseline characteristics categorized by survival status.

**Table 1:** Baseline characteristics of patients by CD4^+^ iATP categories.

Characteristic	High CD4^+^ iATP levels	Low CD4^+^ iATP levels	*p*
	*n* = 40	*n* = 21	
Gender (female *n* (%))	9 (22.50)	1 (4.76)	.157
Age, years	61.95 ± 15.69	51.62 ± 16.68	.020
APACHE II score	17.50 (6.41)	19.19 (7.26)	.354
SOFA score	7.00 [4.75–10.25]	11.00 [8.00–16.00]	.003
CRRT, *n* (%)	11 (27.50)	12 (57.14)	.023
Comorbidity			
Hypertension, *n* (%)	18 (45.00)	3 (14.29)	.016
Diabetes mellitus, *n* (%)	14 (35.00)	5 (23.81)	.370
Liver disease, *n* (%)	4 (10.00)	3 (14.29)	.022
Renal disease, *n* (%)	6 (16.00)	8 (38.10)	.086
Laboratory test			
Lac, mg/dL	1.71 [1.17–1.99]	1.70 [1.27–3.61]	.298
Albumin, g/L	30.20 [27.35–31.85]	30.90 [27.50–33.80]	.271
Bilirubin, μmol/L	16.05 [9.07–25.19]	16.14 [10.88–71.15]	.299
Serum creatinine levels, μmol/L	103.00 [77.50–209.50]	189.00 [110.00–252.00]	.154
CRP, mg/L	130.00 [60.40–222.50]	49.80 [37.90–188.10]	.099
IL‐6, pg/mL	338.30 [71.57–676.38]	48.74 [112.40–412.95]	.270
Procalcitonin, ng/mL	5.32 [1.71–27.66]	5.68 [1.74–24.16]	.938
WBC, ×10^9^/L	11.71 [6.68–16.62]	8.34 [5.10–15.05]	.281
Neutrophil, ×10^9^/L	9.17 [5.42–14.90]	7.28 [4.03–12.76]	.387
Lymphocyte, ×10^9^/L	0.89 [0.56–1.12]	0.56 [0.20–1.15]	.264
CD8^+^ T cell count, ×10^9^/L	0.20 [0.11–0.39]	0.07 [0.02–0.20]	.014
CD4^+^ T cell count, ×10^9^/L	0.31 [0.17–0.44]	0.15 [0.04–0.47]	.13
CD4^+^ iATP, ng/mL	325.54 [219.84–498.80]	61.58 [42.35–97.69]	<.001
CD4^+^ iATP/CD4^+^ T cell count, mg/×10^9^	1276.65 [624.23–2383.32]	593.61 [154.34–1576.91]	.007
Platelets, ×10^9^/L	182.00 [113.50–238.50]	62.00 [30.00–103.00]	<.001
Vital signs			
PaO_2_, mmHg	96 [76.40–142.50]	120 [85.10–178.25]	.092
FIO_2_, %O_2_	0.5 [0.40–0.59]	0.5 [0.39–0.60]	.745
Shock, *n* (%)	12 (30.00)	14 (66.67)	.006
Infection type			
Bacterial, *n* (%)	34 (87.50)	15 (76.19)	.353
Fungal, *n* (%)	5 (12.50)	4 (23.81)	.760
Viral, *n* (%)	0 (0.00)	1 (4.76)	.741
Bacterial + fungal, *n* (%)	1 (2.50）	1 (4.76)	>.999
Mortality rate, *n* (%)	8 (20.0)	13 (61.9)	.001

*Note*: Normally distributed parameters are reported as mean ± standard deviation, while non‐normally distributed parameters are presented as median and interquartile range (IQR; Q1–Q3). Categorical variables are expressed as percentages. Chi‐squared test was employed for variables expressed as percentages. Mann–Whitney *U* test was utilized for those represented as (IQR; Q1–Q3), and Student's *t* test was applied for variables represented as mean ± standard deviation.

Abbreviations: APACHE II, Acute Physiologic Assessment and Chronic Health Evaluation‐II; CRP, C‐reactive protein; CRRT, continuous renal replacement therapy; iATP, intracellular adenosine triphosphate; IL‐6, interleukin‐6; Lac, lactate; LAC, blood lactate level; SOFA, Sequential Organ Failure Assessment score; WBC, white blood cell.

### 3.2 Correlations between CD4^+^ iATP content and other immune‐related parameters

**Figure 2:**
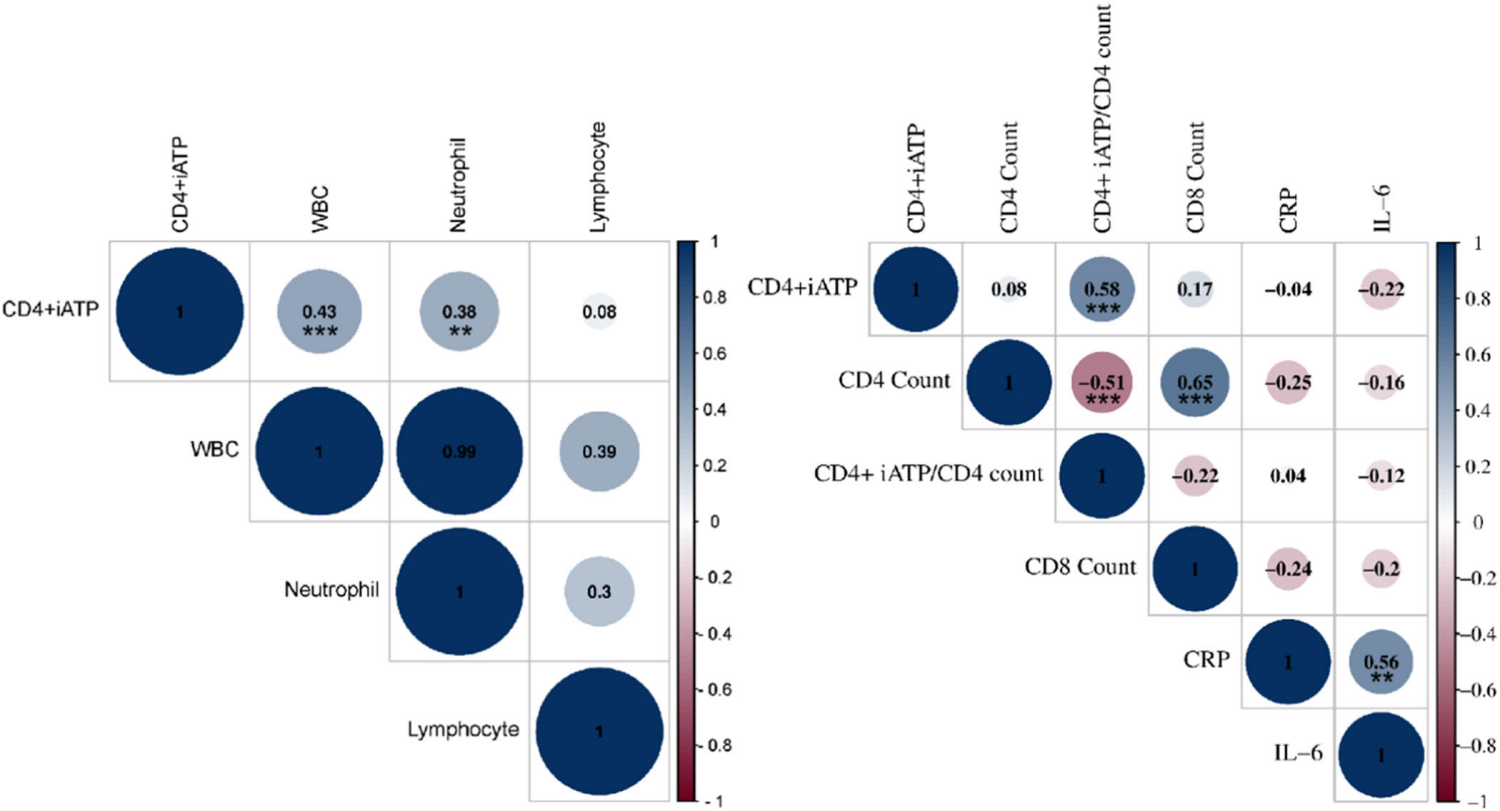
Correlations between CD4^+^ iATP and other immune‐related parameters. Data were presented as circles with different colors. The blue dots, positive correlation; red dots, negative correlation; Pearson correlation coefficient was shown inside the box; ***, *p* < .001; **,*p* < .005. CRP, C‐reactive protein; iATP, intracellular adenosine triphosphate; IL‐6, interleukin‐6; WBC, white blood cell.

In this study, we illuminate the Pearson correlation coefficients that delineate the interconnections between commonly acknowledged immune‐related parameters and the CD4^+^ iATP levels (Figure 2). The results indicate that CD4^+^ iATP levels showed no significant correlations with lymphocyte count, CD4^+^ T cell count, CD8^+^ T cell count, CRP, and IL‐6. However, CD4^+^ iATP content exhibited a positive correlation with WBC count (*r* = .43, *p* < .001), neutrophil count (*r* = .38, *p* = .002), and CD4^+^ iATP/CD4^+^ T cell count ratio (*r* = .58, *p* < .0001).

### 3.3 Associations between CD4^+^ iATP and mortality

**Figure 3:**
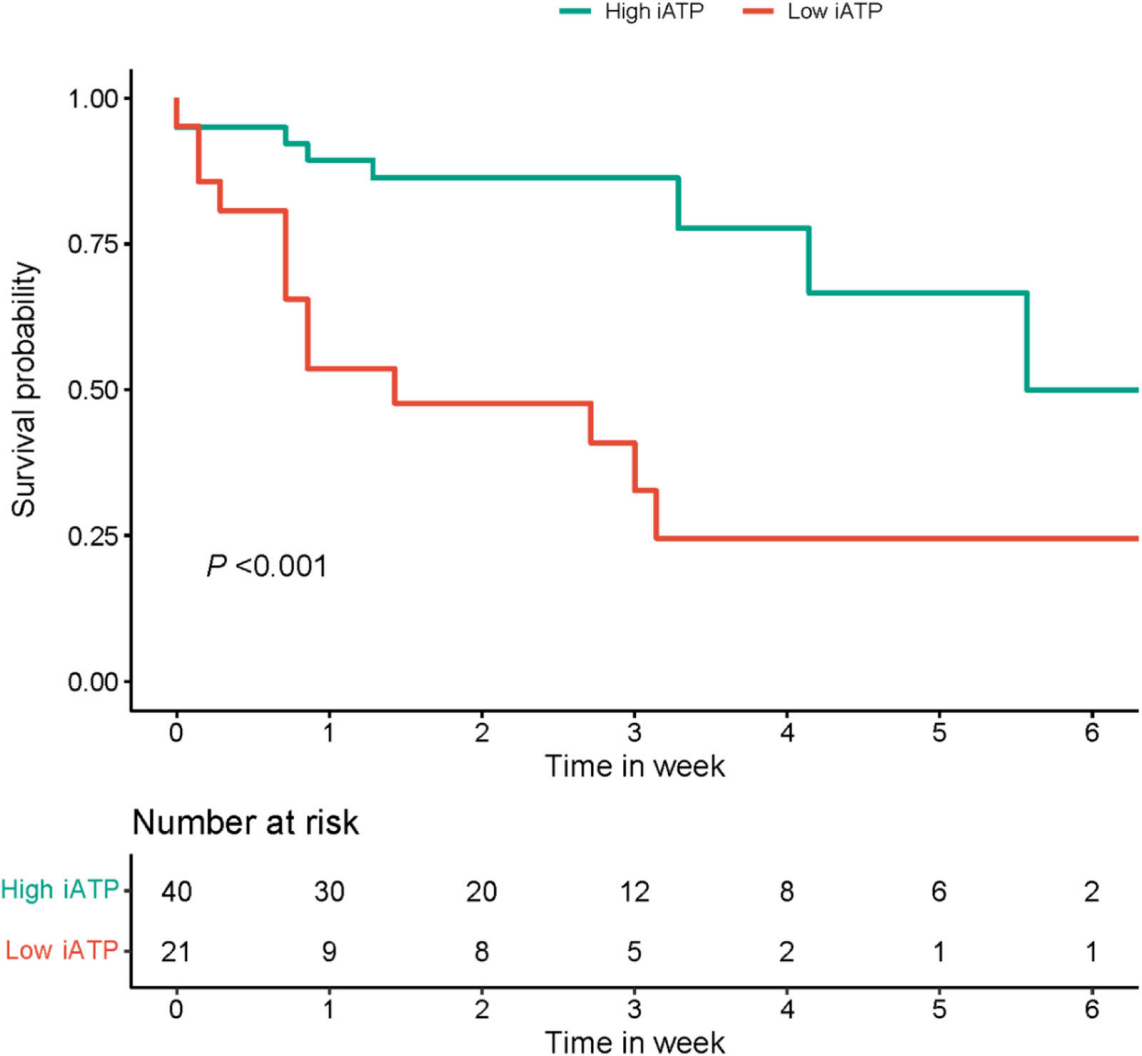
Kaplan–Meier curves for all‐cause mortality by CD4^+^ iATP categories. The comparison of survival rates according to the optimal cut‐off iATP value in high ATP group and low ATP group. iATP, intracellular adenosine triphosphate.

We further investigated the impact of CD4^+^ iATP levels on patient outcomes, we conducted a follow‐up study spanning 28 days. During a follow‐up of 28 days, a total of 21 (34.4%) patients died. The Kaplan–Meier curves based on CD4^+^ iATP categories provide a visual representation of the survival trends, exposing a substantial disparity in the cumulative survival proportions among distinct groups (*p* < .001). This disparity underscores the influence of CD4^+^ iATP levels on the overall survival experience and highlights the potential clinical relevance of this biomarker in predicting patient outcomes (Figure 3). Univariate Cox analyses indicated a statistically significant association between mortality and CD4^+^ iATP categories, SOFA score, APACHE II score, PaO_2_, lactic acid, and presence of septic shock. After adjusting for confounders (SOFA score, APACHE II score, lactic acid, albumin level, and shock rate), patients with low CD4^+^ iATP levels exhibited a 4.5‐fold higher risk of mortality compared to those with high CD4^+^ iATP levels (Model 1: hazard ratio [HR]: 4.515, 95% confidence interval [CI]: 1.276–15.974, *p* = .019). Considering the clinical significance of variables, Model 2, which adjusted for age, gender, SOFA score, and the application of CRRT, revealed that low CD4^+^ iATP levels were indicative of a high risk of mortality, with an HR of 3.512 (95% CI: 1.197–10.306, *p* = .022) (Table 2).

**Table 2:** Univariate and multivariate Cox regression models for CD4^+^ iATP levels and all‐cause mortality.

Characteristic	Univariate			Multivariate Cox analyses					
				Model 1			Model 2		
	HR	95% CI	*p*	HR	95% CI	*p*	HR	95% CI	*p*
CD4^+^ iATP (low vs. high level)	4.626	1.834–11.668	.001	4.515	1.276–15.974	.019	3.512	1.197–10.306	.022
Gender, *n* (%) (female vs. male)	0.584	0.135–2.523	.472				1.09	0.218–5.448	.917
Age, years	0.988	0.964–1.013	.342				1.002	0.974–1.032	.283
APACHE II score	1.072	1.00–1.150	.050	1.00	0.918–1.090	.995			
SOFA score	1.209	1.102–1.327	<.001	1.138	0.990–1.307	.069	1.192	1.072–1.326	.001
CRRT, *n* (%) (yes vs. no)	1.578	0.654–3.806	.310				0.551	0.185–1.637	.283
Comorbidity									
Hypertension, *n* (%) (yes vs. no)	0.306	0.089–1.047	.059						
Diabetes mellitus, *n* (%) (yes vs. no)	1.114	0.444–2.798	.818						
Liver disease, *n* (%) (yes vs. no)	2.458	0.812–7.440	.112						
Renal disease, n (%) (yes vs. no)	1.705	0.677–4.293	.250						
Laboratory test									
Lac, mg/dL	1.206	1.095–1.329	<.001	1.041	0.904–1.199	.574			
Albumin, g/L	0.992	0.915–1.076	.849	0.944	0.859–1.038	.235			
Bilirubin, μmol/L	1.003	0.999–1.007	.091						
Serum creatinine levels, μmol/L	1.003	1.000–1.006	.072						
CRP, mg/L	0.999	0.995–1.004	.814						
IL‐6, pg/mL	0.999	0.999–1.000	.104						
Procalcitonin, ng/mL	1.007	0.996–1.019	.215						
WBC, ×10^9^/L	0.976	0.927–1.027	.344						
Neutrophil, ×10^9^/L	0.977	0.925–1.031	.394						
Lymphocyte, ×10^9^/L	0.623	0.285–1.361	.235						
CD8^+^ T cell count, ×10^9^/L	1.067	0.485–2.347	.872						
CD4^+^ T cell count, ×10^9^/L	0.266	0.018–3.963	.337						
CD4^+^ iATP/CD4^+^ T cell count, mg/×10^9^	1.000	0.999–1.000	.294						
Platelets, ×10^9^/L	0.990	0.983–0.997	.004						
Vital signs									
PaO_2_, mmHg	1.01	1.000–1.019	.049						
FIO_2_, mmHg	23.357	0.491–1112.017	.110						
Shock, *n* (%) (yes vs. no)	3.235	1.236–8.467	.017	1.012	0.304–3.375	.984			
Infection type									
Bacterial, *n* (%)	Reference	Reference	Reference						
Fungal, *n* (%)	0.624	0.177–2.196	.462						
Viral, *n* (%)	0.000	0.000	.986						
Bacterial + fungal, *n* (%)	2.151	0.279–16.569	.402						

*Note*: Model I includes CD4^+^ iATP, APACHE II score, SOFA score, lactic acid, albumin level, and presence of septic shock; Model II includes CD4^+^ iATP, age, gender, SOFA score, and the application of CRRT.

Abbreviations: APACHE II, Acute Physiologic Assessment and Chronic Health Evaluation‐II; CRP, C‐reactive protein; CRRT, continuous renal replacement therapy; iATP, intracellular adenosine triphosphate; Lac, blood lactate level; SOFA, Sequential Organ Failure Assessment score; WBC, white blood cell.

## 4 DISCUSSION

The measurement of iATP has been shown to provide a systemic representation of CD4^+^ T cell function.^32^ Our study demonstrated that low levels of CD4^+^ iATP were associated with an increased risk of death. Through the incorporation of various models, including SOFA score, APACHE II score, lactic acid, albumin level, and shock rate, we consistently observed that patients with CD4^+^ iATP levels below 132.24 ng/mL had a three to five times higher risk of mortality compared to those with CD4^+^ iATP levels above this threshold (Table 2). The average CD4^+^ iATP level among non‐survivors was significantly lower than the normal range.^27^ The lower CD4^+^ iATP levels in non‐survivors compared to survivors were associated with the 28‐day mortality rate in sepsis patients (Figure 3). Hence, monitoring CD4^+^ iATP levels during the early stages of sepsis may prove beneficial for risk assessment in sepsis patients.

The underlying reason for the observed association between lower CD4^+^ iATP levels and a poorer prognosis in sepsis patients remains uncertain, necessitating further investigation. Nevertheless, there are several potential explanations that warrant exploration. First, this decline in ATP content could be attributed to mitochondrial dysfunction, which can impair ATP production.^33^, ^34^ Second, it may be linked to lymphocyte anergy, a common characteristic of immune dysfunction in sepsis patients, resulting in reduced responsiveness to mitogen stimulation.^35^, ^36^, ^37^ It is worth noting that studies have reported a switch from oxidative phosphorylation (OXPHOS) to aerobic glycolysis as a hallmark of T cell activation, enabling them to meet the metabolic demands of proliferation.^38^ Additionally, it is plausible that this decrease may signify lymphocyte bioenergetic failure, representing an additional form of organ dysfunction within the context of sepsis. Further research is required to elucidate the precise mechanisms underlying this association and provide a comprehensive understanding of the relationship between CD4^+^ iATP content and prognosis in sepsis patients.

The lack of correlation between CD4^+^ iATP and CD4^+^ T cell or lymphocyte count observed in our study is an interesting finding (Figure 2), as it suggests that these two parameters may reflect different aspects of immune function. While CD4^+^ T cell count is a widely used measure of immune health, it may not fully capture the functional state of CD4^+^ T cells, as their activation and metabolic status can vary independently of their total count.^38^, ^39^ The activation and metabolic status of CD4^+^ T cells can vary independently of their total count, and their capacity to synthesize and store ATP may also differ depending on their activation state and metabolic demands.^40^ Another intriguing observation is the close correlation between CD4^+^ iATP levels and mortality in sepsis, whereas isolated CD4^+^ T cell count or CD4^+^ iATP/CD4^+^ T cell count do not correlate with sepsis mortality. CD4^+^ iATP levels studied in our research actually result from a combination of cell quantity and individual CD4^+^T cell ATP levels. We speculate that a single index may not fully represent a patient's CD4 cell immune function, as quantity and individual cell ATP levels can complement each other. Many patients exhibit insufficient CD4^+^ T cell count, but sufficient individual cell ATP levels, or vice versa, leading to maintenance of overall immune function within normal ranges. However, the specific mechanism behind this phenomenon requires further exploration.

Our study revealed a significant correlation between CD4^+^ iATP concentration and the counts of white blood cells and neutrophils in sepsis patients, consistent with the findings reported by Naderi et al.^26^, ^41^ However, a previous study indicated no association between iATP levels and white blood cell counts.^32^ Therefore, further research is needed to determine whether CD4+ iATP levels can effectively reflect systemic inflammation and innate immune responses.

It is important to note that iATP has been demonstrated to systematically represent the overall function of CD4^+^ T cells.^42^ The ATP measurement conducted using the PHA‐L as a mitogen, which selectively activates CD4^+^ T cells.^32^ These findings suggest that iATP concentration values may serve as a reliable indicator of CD4^+^ T cell function and provide insights into the immune response in sepsis patients.

The understanding of sepsis has evolved to recognize the involvement of both pro‐inflammatory and anti‐inflammatory phases at different stages of the disease. Consequently, immunotherapy during sepsis has emerged as a significant area of research worldwide. Achieving optimal dosing of immunomodulatory drugs is crucial for improving patient outcomes, and numerous randomized controlled trials have explored the use of immunomodulatory therapies (e.g., GM‐CSF, Tymosin α1, and IL‐7) for sepsis treatment.^43^, ^44^, ^45^ In addition to these therapies, monitoring immune markers such as mHLA‐DR and absolute lymphocyte counts has been investigated as a means of identifying septic patients with immunosuppression and guiding treatment decisions. However, there are still some studies that found that the predictive power of the HLA receptors has yielded mixed results. They were unable to find a good correlation between mHLA‐DR downregulation or lymphocyte apoptosis and mortality when adjusted for confounders.^46^, ^47^ Looking for reliable pro‐inflammatory or anti‐inflammatory markers to evaluate the immune state is an urgent task.

Our study, along with other relevant reports, supports the notion that monitoring the concentrations of iATP produced by CD4^+^ T cells could be valuable in distinguishing between hypoimmune and hyperimmune states.^26^ This approach may aid in identifying patients at risk of infection or death, adjusting immunosuppressive agents according to CD4^+^ iATP content can help reduce mortality and infection in transplant patients, ultimately enhancing the effectiveness of immunotherapy interventions. By assessing CD4^+^ iATP levels, clinicians may be able to stratify patients and tailor immunomodulatory treatments accordingly, optimizing their response to therapy. This highlights the potential of iATP monitoring as a valuable tool in the management of sepsis‐induced immunosuppression.

Our study has several limitations that should be acknowledged. First, the small sample size might introduce potential statistical biases or random errors. Additionally, our study was conducted at a single medical center and within a single ethnic group, which may limit the generalizability of the findings to other geographic locations, populations, or cultural settings. Despite adjusting for SOFA and other relevant indicators, there might be other unconsidered confounding factors that could influence the results. Furthermore, our study primarily focused on the relationship between CD4^+^ iATP and mortality and did not explore its association with other important clinical outcomes such as disease severity or length of hospital stay. This limitation restricts our comprehensive understanding and application of CD4^+^ iATP in the context of sepsis.

To gain a deeper understanding of the mechanisms underlying the association between CD4^+^ iATP content and sepsis prognosis, further research is warranted. This could involve designing multicenter randomized controlled trials to investigate the impact of immunomodulatory therapy on immune function and assessing the effects of changes in immune function on various clinical outcomes, including mortality, acute kidney injury, and duration of hospitalization. Such studies would contribute to advancing our knowledge and enhancing the clinical application of iATP monitoring in sepsis management.

### 4.1 Conclusion

Our study demonstrates a strong association between low CD4^+^ iATP levels and increased mortality in sepsis patients. These findings highlight the potential of CD4^+^ iATP as a prognostic marker in sepsis management. To further validate its clinical utility, prospective clinical trials are warranted to evaluate the efficacy of CD4^+^ iATP‐based immunologic interventions on patient outcomes. Such trials would provide valuable insights into the effectiveness of interventions targeting CD4^+^ iATP levels in improving outcomes for sepsis patients.

## AUTHOR CONTRIBUTIONS

Conceptualization: Kouxing Zhang. Formal analysis: Changlong Zheng and Min Fan. Project administration: Ying Xian. Visualization: Kefeng Jiang. Writing—original draft: Ying Xian and Dan Xie. Writing—review and editing: Kouxing Zhang.

## CONFLICT OF INTEREST STATEMENT

The authors declare no conflict of interest.

## ETHICS STATEMENT

This study was approved by the ethical committee of the Third Affiliated Hospital of Sun Yat‐sen University (No. [2022]02‐216‐01). All participants in this study provided written informed consent, allowing their clinical data to be utilized. The study adhered to the principles outlined in the Declaration of Helsinki.

## Supporting information

Supporting information.

## Data Availability

The data sets produced and evaluated during the present study cannot be accessed by the public due to privacy constraints but can be obtained from the corresponding author upon making a reasonable request.

## References

[1] Xie J , Wang H , Kang Y , et al. The epidemiology of sepsis in Chinese ICUs: a national cross‐sectional survey. Crit Care Med. 2020;48(3):e209‐e218. 31804299 10.1097/CCM.0000000000004155

[2] Liu Y‐C , Yao Y , Yu M‐M , et al. Frequency and mortality of sepsis and septic shock in China: a systematic review and meta‐analysis. BMC Infect Dis. 2022;22(1):564. 35729526 10.1186/s12879-022-07543-8 PMC9210671

[3] Topkan E , Selek U , Ozdemir Y , et al. Prognostic value of the Glasgow Prognostic Score for glioblastoma multiforme patients treated with radiotherapy and temozolomide. J Neurooncol. 2018;139:411‐419. 29696530 10.1007/s11060-018-2879-4

[4] Akavipat P . Acute physiology and chronic health evaluation (APACHE) II score—the clinical predictor in neurosurgical intensive care unit. Acta Clin Croat. 2019;58(1):50. 31363325 10.20471/acc.2019.58.01.07 PMC6629196

[5] Hotchkiss RS , Monneret G , Payen D . Sepsis‐induced immunosuppression: from cellular dysfunctions to immunotherapy. Nat Rev Immunol. 2013;13(12):862‐874. 24232462 10.1038/nri3552 PMC4077177

[6] Pei F , Yao R‐Q , Ren C , et al. Expert consensus on the monitoring and treatment of sepsis‐induced immunosuppression. Mil Med Res. 2022;9(1):74. 36567402 10.1186/s40779-022-00430-y PMC9790819

[7] Chihade DB , Smith P , Swift DA , et al. Myosin light chain kinase deletion worsens lung permeability and increases mortality in pneumonia‐induced sepsis. Shock. 2023;59(4):612‐620. 36640152 10.1097/SHK.0000000000002081 PMC10065930

[8] Wilson JK , Zhao Y , Singer M , Spencer J , Shankar‐Hari M . Lymphocyte subset expression and serum concentrations of PD‐1/PD‐L1 in sepsis‐pilot study. Crit Care. 2018;22(1):95. 29661225 10.1186/s13054-018-2020-2 PMC5902875

[9] Wu T , Ren C , Dou X , et al. Interleukin‐35 downregulates the immune response of effector CD4+ T cells via restricting high mobility group box‐1 protein‐dependent autophagy in sepsis. Shock. 2023;59(2):277‐287. 36731088 10.1097/SHK.0000000000001990

[10] Hotchkiss RS , Swanson PE , Freeman BD , et al. Apoptotic cell death in patients with sepsis, shock, and multiple organ dysfunction. Crit Care Med. 1999;27(7):1230‐1251. 10446814 10.1097/00003246-199907000-00002

[11] Chang K , Svabek C , Vazquez‐Guillamet C , et al. Targeting the programmed cell death 1: programmed cell death ligand 1 pathway reverses T cell exhaustion in patients with sepsis. Crit Care. 2014;18:R3. 24387680 10.1186/cc13176 PMC4056005

[12] Muszynski JA , Nofziger R , Greathouse K , et al. Early adaptive immune suppression in children with septic shock: a prospective observational study. Crit Care. 2014;18:R145. 25005517 10.1186/cc13980 PMC4226962

[13] Shao R , Li C‐S , Fang Y , Zhao L , Hang C . Low B and T lymphocyte attenuator expression on CD4+ T cells in the early stage of sepsis is associated with the severity and mortality of septic patients: a prospective cohort study. Crit Care. 2015;19:308. 26329820 10.1186/s13054-015-1024-4 PMC4556404

[14] Misra AK , Levy MM , Ward NS . Biomarkers of immunosuppression. Crit Care Clin. 2020;36(1):167‐176. 31733678 10.1016/j.ccc.2019.08.013

[15] Pfortmueller CA , Meisel C , Fux M , Schefold JC . Assessment of immune organ dysfunction in critical illness: utility of innate immune response markers. Intensive Care Med Exp. 2017;5:49. 29063386 10.1186/s40635-017-0163-0 PMC5653680

[16] Lindell RB , Zhang D , Bush J , et al. Impaired lymphocyte responses in pediatric sepsis vary by pathogen type and are associated with features of immunometabolic dysregulation. Shock. 2022;57(6):191‐199. 35759301 10.1097/SHK.0000000000001943 PMC9245144

[17] Heffernan DS , Chung C‐S , Ayala A . Splenic invariant natural killer T cells play a significant role in the response to polymicrobial sepsis. Shock. 2023;60(3):443‐449. 37493576 10.1097/SHK.0000000000002185 PMC10529630

[18] Hotchkiss RS , Osmon SB , Chang KC , Wagner TH , Coopersmith CM , Karl IE . Accelerated lymphocyte death in sepsis occurs by both the death receptor and mitochondrial pathways. J Immunol. 2005;174(8):5110‐5118. 15814742 10.4049/jimmunol.174.8.5110

[19] Weiss SL , Zhang D , Bush J , et al. Mitochondrial dysfunction is associated with an immune paralysis phenotype in pediatric sepsis. Shock. 2020;54(3):285‐293. 31764621 10.1097/SHK.0000000000001486 PMC7325426

[20] Weiss SL , Zhang D , Bush J , et al. Persistent mitochondrial dysfunction linked to prolonged organ dysfunction in pediatric sepsis. Crit Care Med. 2019;47(10):1433‐1441. 31385882 10.1097/CCM.0000000000003931 PMC7341116

[21] Weiss SL , Selak MA , Tuluc F , et al. Mitochondrial dysfunction in peripheral blood mononuclear cells in pediatric septic shock. Pediatr Crit Care Med. 2015;16(1):e4. 25251517 10.1097/PCC.0000000000000277 PMC4286436

[22] Monneret G , Lepape A , Voirin N , et al. Persisting low monocyte human leukocyte antigen‐DR expression predicts mortality in septic shock. Intensive Care Med. 2006;32:1175‐1183. 16741700 10.1007/s00134-006-0204-8

[23] De Roquetaillade C , Dupuis C , Faivre V , Lukaszewicz AC , Brumpt C , Payen D . Monitoring of circulating monocyte HLA‐DR expression in a large cohort of intensive care patients: relation with secondary infections. Ann Intensive Care. 2022;12(1):39. 35526199 10.1186/s13613-022-01010-y PMC9079217

[24] Joshi I , Carney WP , Rock EP . Utility of monocyte HLA‐DR and rationale for therapeutic GM‐CSF in sepsis immunoparalysis. Front Immunol. 2023;14:1130214. 36825018 10.3389/fimmu.2023.1130214 PMC9942705

[25] Kobashigawa JA , Kiyosaki KK , Patel JK , et al. Benefit of immune monitoring in heart transplant patients using ATP production in activated lymphocytes. J Heart Lung Transplant. 2010;29(5):504‐508. 20133166 10.1016/j.healun.2009.12.015

[26] Xue F , Gao W , Qin T , et al. Immune cell function assays in the diagnosis of infection in pediatric liver transplantation: an open‐labeled, two center prospective cohort study. Transl Pediatr. 2021;10(2):333‐343. 33708519 10.21037/tp-20-256 PMC7944184

[27] Lawrence KL , White PH , Morris GP , et al. CD4+ lymphocyte adenosine triphosphate determination in sepsis: a cohort study. Crit Care. 2010;14:R110. 20540723 10.1186/cc9059 PMC2911756

[28] Shankar‐Hari M , Phillips GS , Levy ML , et al. Developing a new definition and assessing new clinical criteria for septic shock: for the Third International Consensus Definitions for Sepsis and Septic Shock (Sepsis‐3). JAMA. 2016;315(8):775‐787. 26903336 10.1001/jama.2016.0289 PMC4910392

[29] Singer M , Deutschman CS , Seymour CW , et al. The third international consensus definitions for sepsis and septic shock (Sepsis‐3). JAMA. 2016;315(8):801‐810. 26903338 10.1001/jama.2016.0287 PMC4968574

[30] Vincent J‐L , Moreno R , Takala J , et al. The SOFA (Sepsis‐related Organ Failure Assessment) Score To Describe Organ Dysfunction/failure: On Behalf of the Working Group on Sepsis‐Related Problems of the European Society of Intensive Care Medicine (see Contributors to the Project in the Appendix). Springer‐Verlag; 1996. 10.1007/BF01709751 8844239

[31] Knaus WA , Draper EA , Wagner DP , Zimmerman JE . APACHE II: a severity of disease classification system. Crit Care Med. 1985;13(10):818‐829. 3928249

[32] Sugiyama K , Tsukaguchi M , Sasahara H , et al. Relationship between the peripheral lymphocyte response to mycophenolic acid in vitro and the level of ATP in peripheral CD4+ lymphocytes before and after renal transplantation. Drug Res. 2014;65:629‐634. 10.1055/s-0034-1395686 25549254

[33] Yang H , Zhang Z . Sepsis‐induced myocardial dysfunction: the role of mitochondrial dysfunction. Inflamm Res. 2021;70:379‐387. 33683374 10.1007/s00011-021-01447-0

[34] Gatza E , Wahl DR , Opipari AW , et al. Manipulating the bioenergetics of alloreactive T cells causes their selective apoptosis and arrests graft‐versus‐host disease. Sci Transl Med. 2011;3(67):67ra8‐6ra8ra8. 10.1126/scitranslmed.3001975 PMC3364290 21270339

[35] Ledo C , Gonzalez CD , Poncini CV , Mollerach M , Gómez MI . TNFR1 signaling contributes to T cell anergy during *Staphylococcus aureus* sepsis. Front Cell Infect Microbiol. 2018;8:259. 30123776 10.3389/fcimb.2018.00259 PMC6085448

[36] Wang A , Zhang S , Peng G , Tang Y , Yang Y . ICU and sepsis: role of myeloid and lymphocyte immune cells. J Oncol. 2022;2022:1‐7. 10.1155/2022/7340266 PMC9527402 36199798

[37] Gerriets VA , Rathmell JC . Metabolic pathways in T cell fate and function. Trends Immunol. 2012;33(4):168‐173. 22342741 10.1016/j.it.2012.01.010 PMC3319512

[38] Chang CH , Curtis JD , Maggi LB , et al. Posttranscriptional control of T cell effector function by aerobic glycolysis. Cell. 2013;153(6):1239‐1251. 23746840 10.1016/j.cell.2013.05.016 PMC3804311

[39] Rodríguez‐Pascual F , Redondo‐Horcajo M , Magán‐Marchal N , et al. Glyceraldehyde‐3‐phosphate dehydrogenase regulates endothelin‐1 expression by a novel, redox‐sensitive mechanism involving mRNA stability. Mol Cell Biol. 2008;28(23):7139‐7155. 18809573 10.1128/MCB.01145-08 PMC2593384

[40] Kolan SS , Li G , Wik JA , et al. Cellular metabolism dictates T cell effector function in health and disease. Scand J Immunol. 2020;92:e12956. 32767795 10.1111/sji.12956

[41] Naderi H , Pourmand G , Dehghani S , Nikoueinejad H , Jafari M , Tajik N . Monitoring cellular immune function of renal transplant recipients based on adenosine triphosphate (ATP) production by mitogen‐induced CD4+ T helper cells. Biomed Pharmacother. 2018;107:1402‐1409. 30257356 10.1016/j.biopha.2018.08.110

[42] Lenherr N , Christodoulou J , Duley J , et al. Co‐therapy with S‐adenosylmethionine and nicotinamide riboside improves t‐cell survival and function in Arts Syndrome (PRPS1 deficiency). Mol Genet Metab Rep. 2021;26:100709. 33532242 10.1016/j.ymgmr.2021.100709 PMC7823043

[43] Francois B , Jeannet R , Daix T , et al. Interleukin‐7 restores lymphocytes in septic shock: the IRIS‐7 randomized clinical trial. JCI Insight. 2018;3(5):e98960. 29515037 10.1172/jci.insight.98960 PMC5922293

[44] Liu F , Wang H‐M , Wang T , Zhang Y‐M , Zhu X . The efficacy of thymosin α1 as immunomodulatory treatment for sepsis: a systematic review of randomized controlled trials. BMC Infect Dis. 2016;16(1):488. 27633969 10.1186/s12879-016-1823-5 PMC5025565

[45] Pinder EM , Rostron AJ , Hellyer TP , et al. Randomised controlled trial of GM‐CSF in critically ill patients with impaired neutrophil phagocytosis. Thorax. 2018;73(10):918‐925. 30064991 10.1136/thoraxjnl-2017-211323 PMC6166597

[46] Gomez HG , Gonzalez SM , Londoño JM , et al. Immunological characterization of compensatory anti‐inflammatory response syndrome in patients with severe sepsis: a longitudinal study. Crit Care Med. 2014;42(4):771‐780. 24365860 10.1097/CCM.0000000000000100

[47] Perry SE , Mostafa SM , Wenstone R , Shenkin A , McLaughlin PJ . Is low monocyte HLA‐DR expression helpful to predict outcome in severe sepsis? Intensive Care Med. 2003;29(8):1245‐1252. 12774155 10.1007/s00134-003-1686-2

