# Supplementary material for: Association between intracellular adenosine triphosphate content of CD4+ T lymphocytes and mortality in sepsis patients: A prospective observational study
Source: Immun Inflamm Dis. 2024 Jun 11;12(6):e1286. doi: 10.1002/iid3.1286 (PMC11165683; doi:10.1002/iid3.1286)
Supplement: Supplementary file 1 — Supporting information. [file IID3-12-e1286-s001.docx]

Supplementary Material

**Association between intracellular adenosine triphosphate content of CD4^+^ T lymphocytes and mortality in sepsis patients:A prospective observational study**

**Ying Xian^1^†, Dan Xie^1^†, Jian Zhu^2^, Changlong Zheng^2^, Min Fan^1^, Kefeng Jiang^3^,Kouxing Zhang^1^***

*** Correspondence:** Kouxing Zhang: [zhkoux@mail.sysu.edu.cn](mailto:zhkoux@mail.sysu.edu.cn)

**Table S1. Baseline characteristics of patients grouped by survival status**

| Characteristic | **Alive** | **Dead** | *P* |
| --- | --- | --- | --- |
|  | n=40 | n=21 |  |
| **Gender, (female n (%))** | 7 (17.50) | 3 (14.29%) | >0.999 |
| **Age, years** | 61.00±16.21 | 53.43±16.73 | 0.092 |
| **APACHEII score** | 16.52 (6.57) | 21.05 (6.05) | 0.011 |
| **SOFA score** | 7.00 [4.00, 10.00] | 11.00 [8.00, 16.00] | <0.001 |
| **CRRT,n(%)** | 12 (30.00) | 11 (52.38) | 0.151 |
| **Comorbidity** |  |  |  |
| Hypertension, n(%) | 17 (42.50) | 4 (19.05) | 0.122 |
| Diabetes mellitus, n(%) | 12 (30.00) | 7 (33.33) | >0.999 |
| Liver disease, n(%) | 3(7.5) | 4(19.05) | 0.145 |
| Renal disease, n(%) | 7(17.50) | 7(33.33) | 0.118 |
| **Laboratory test** |  |  |  |
| Lac, mg/dL | 1.64 [1.13, 1.88] | 2.11 [1.29, 4.56] | 0.033 |
| Albumin, g/L | 30.50 [27.60, 32.40] | 30.40 [24.65, 34.15] | 0.944 |
| Bilirubin, μmol/L | 13.04 [7.79, 22.15] | 30.86 [12.48, 93.88] | 0.004 |
| Serum creatinine levels, μmol/L | 101.00 [69.00, 213.00] | 167.00 [107.50, 277.00] | 0.038 |
| CRP, mg/L | 89.70[47.10, 208.20] | 136.10 [44.90, 237.20] | 0.802 |
| IL-6, Pg/ml | 177.45[52.39-500.23] | 208.60[107.80-2145.00] | 0.051 |
| Procalcitonin, ng/mL | 6.90 [1.64, 25.36] | 3.97 [2.11, 63.90] | 0.919 |
| WBC, ×10^9^/L | 10.91 [7.03, 16.19] | 8.45 [5.10, 15.05] | 0.295 |
| Neutrophil, ×10^9^/L | 8.75 [5.42, 13.74] | 7.28 [3.82, 14.65] | 0.43 |
| Lymphocyte, ×10^9^/L | 0.89 [0.56, 1.30] | 0.56 [0.20, 0.99] | 0.045 |
| CD8^+^ T cell Count, ×10^9^/L | 0.18[0.11, 0.39] | 0.07[0.02, 0.31] | 0.043 |
| CD4^+^ T cell Count, ×10^9^/L | 0.30[0.15, 0.44] | 0.22 [0.06,0.41] | 0.242 |
| CD4^+^ iATP, ng/ml | 265.69 [142.36, 407.78] | 100.78 [46.81, 347.89] | 0.01 |
| CD4^+^ iATP/CD4^+^ T cell Count, mg/×10^9^ | 1110.79[574.69, 2192.19] | 751.35[525.81, 1922.35] | 0.408 |
| Platelets, ×10^9^/L | 174.00 [100.00, 241.00] | 65.00 [27.00, 106.00] | <0.001 |
| **Vital signs** |  |  |  |
| PaO2, mmHg | 98.95[76.40,143.23] | 97.80[84.80,178.25] | 0.202 |
| FIO2, %O2 | 0.48[0.37,0.52] | 0.5[0.50,0.60] | 0.024 |
| Shock, n(%) | 12(30.00) | 14(66.67) | 0.003 |
| **Infection type** |  |  |  |
| Bacterial, n(%) | 29(72.50) | 17(80.95) | 0.466 |
| Fungal, n(%) | 6(15.00) | 4(23.81) | 0.655 |
| Viral, n(%) | 1(2.50) | 0(0.00) | 0.471 |
| Bacterial+fungal, n(%) | 1(2.50） | 1(4.76) | >0.999 |

**Abbreviations：**CRRT, continuous renal replacement therapy; LAC, blood lactate level; APACHE II, Acute Physiologic Assessment and Chronic Health Evaluation-II; SOFA, sequential organ failure assessment score; Lac, Lactate; CRP, C-reactive protein; IL-6, interleukin-6; iATP intracellular adenosine triphosphate; WBC, white blood cell.

Normally distributed parameters are reported as mean±standard deviation, while non-normally distributed parameters are presented as median and interquartile range (IQR; Q1–Q3). Categorical variables are expressed as percentages. Chi-squared test was employed for variables expressed as percentages. Mann–Whitney U test was utilized for those represented as (IQR; Q1–Q3), and Student's t-test was applied for variables represented as mean±standard deviation.


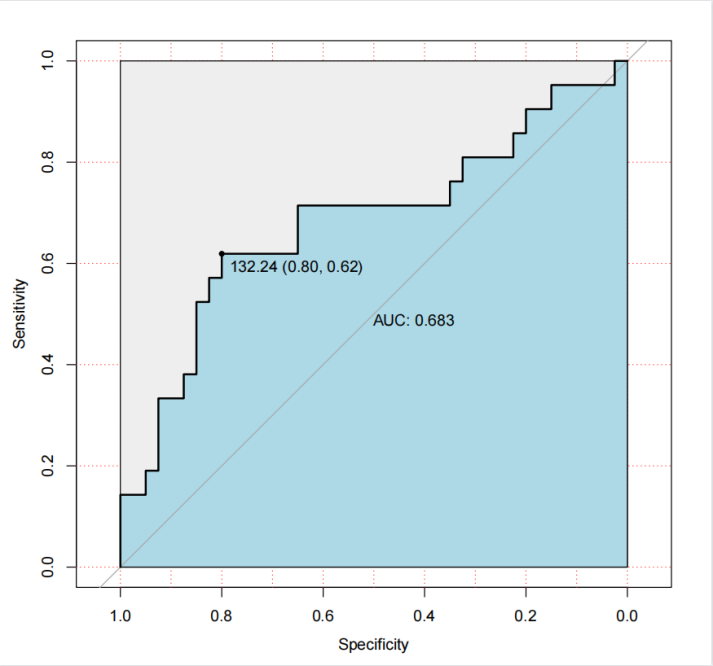


**Figure S1. Receiver operating characteristic curves of CD4^+^ iATP for predicting mortality.**

Area under the curve (AUC) was 0.683. The optimal cut-off value was 132.24ng/ml
